# Association of SARS-CoV-2 Load in Wastewater With Reported COVID-19 Cases in the Tokyo 2020 Olympic and Paralympic Village From July to September 2021

**DOI:** 10.1001/jamanetworkopen.2022.26822

**Published:** 2022-08-22

**Authors:** Masaaki Kitajima, Michio Murakami, Syun-suke Kadoya, Hiroki Ando, Tomohiro Kuroita, Hiroyuki Katayama, Seiya Imoto

**Affiliations:** 1 Division of Environmental Engineering, Faculty of Engineering, Hokkaido University, Kita-ku, Sapporo, Hokkaido, Japan; 2 Center for Infectious Disease Education and Research, Osaka University, Suita, Osaka, Japan; 3 Department of Urban Engineering, Graduate School of Engineering, The University of Tokyo, Bunkyo-ku, Tokyo, Japan; 4 Shionogi & Co Ltd, Chuo-ku, Osaka, Osaka, Japan; 5 Human Genome Center, The Institute of Medical Science, The University of Tokyo, Minato-ku, Tokyo, Japan

## Abstract

This cross-sectional study assesses the association between SARS-CoV-2 load in wastewater and confirmed cases of COVID-19 in the Tokyo 2020 Olympic and Paralympic Village.

## Introduction

Because SARS-CoV-2 transmission was a major concern during the Tokyo 2020 Olympic and Paralympic Games, wastewater surveillance,^1^ mandatory daily screenings with antigen saliva tests,^2^ and polymerase chain reaction (PCR) tests for close contacts of individuals with confirmed cases^3^ were conducted in the Olympic and Paralympic Village. In this cross-sectional study, we investigated the association of SARS-CoV-2 load in wastewater with the numbers of confirmed COVID-19 cases and tests for close contacts.

## Methods

From July 14 through September 8, 2021, 360 wastewater samples were collected via passive sampling from manholes in 7 distinct areas of the village and examined for SARS-CoV-2 RNA using quantitative PCR.^4^ Wastewater sampling, SARS-CoV-2 RNA analysis, and data reporting to the Organizing Committee for the Olympic and Paralympic Games were performed daily. The numbers of confirmed COVID-19 cases and close contacts tests were obtained from the committee and a recent report,^3^ respectively. This study followed the STROBE reporting guideline. Informed consent and ethics approval were not required because this study was outside the scope of ethical guidelines set by the Ministry of Education, Culture, Sports, Science and Technology, Japan. Statistical analyses were performed with SPSS, version 28. One- or 2-tailed *P* = .05 was considered significant. Details are given in the eMethods in the Supplement.

## Results

**Figure 1.**
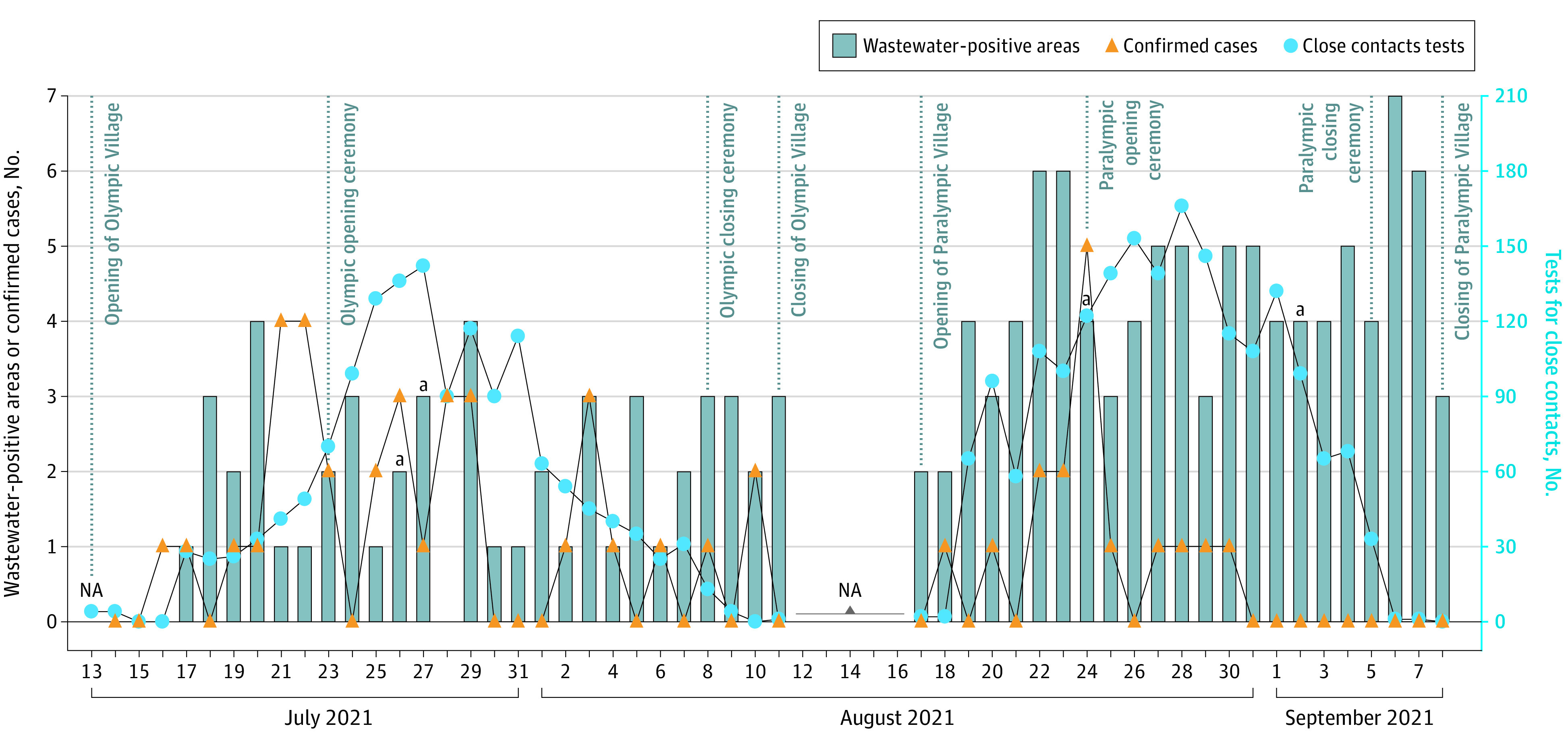
Detection of SARS-CoV-2 RNA From Wastewater in the Tokyo 2020 Olympic and Paralympic Village Data on close contacts tests are from Akashi et al.^3^ The number of confirmed COVID-19 cases, for which information was publicly available on the Tokyo 2020 Olympic and Paralympics website during the Olympic and Paralympic Games, was provided by the Organizing Committee for the Olympic and Paralympic Games. NA indicates no available wastewater sample. ^a^Wastewater sample was not available from 1 of the 7 areas.

The village accommodated approximately 11 000 and 4400 participants during the Olympics and Paralympics, respectively.^3^ SARS-CoV-2 RNA was detected in 151 wastewater samples (41.9%), of which 53 (26.4%) and 98 (61.6%) were from the Olympics and Paralympics, respectively (Figure 1), indicating a significantly higher positivity rate in the latter (φ = 0.35; *P* < .001, 2-tailed χ^2^ test). The numbers of confirmed cases and close contact tests per participant were higher during the Paralympics than during the Olympics (3.2 vs 3.6 confirmed cases per 1000 participants; 140 vs 440 close contacts tests per 1000 participants^3^) (Figure 1).

**Figure 2.**
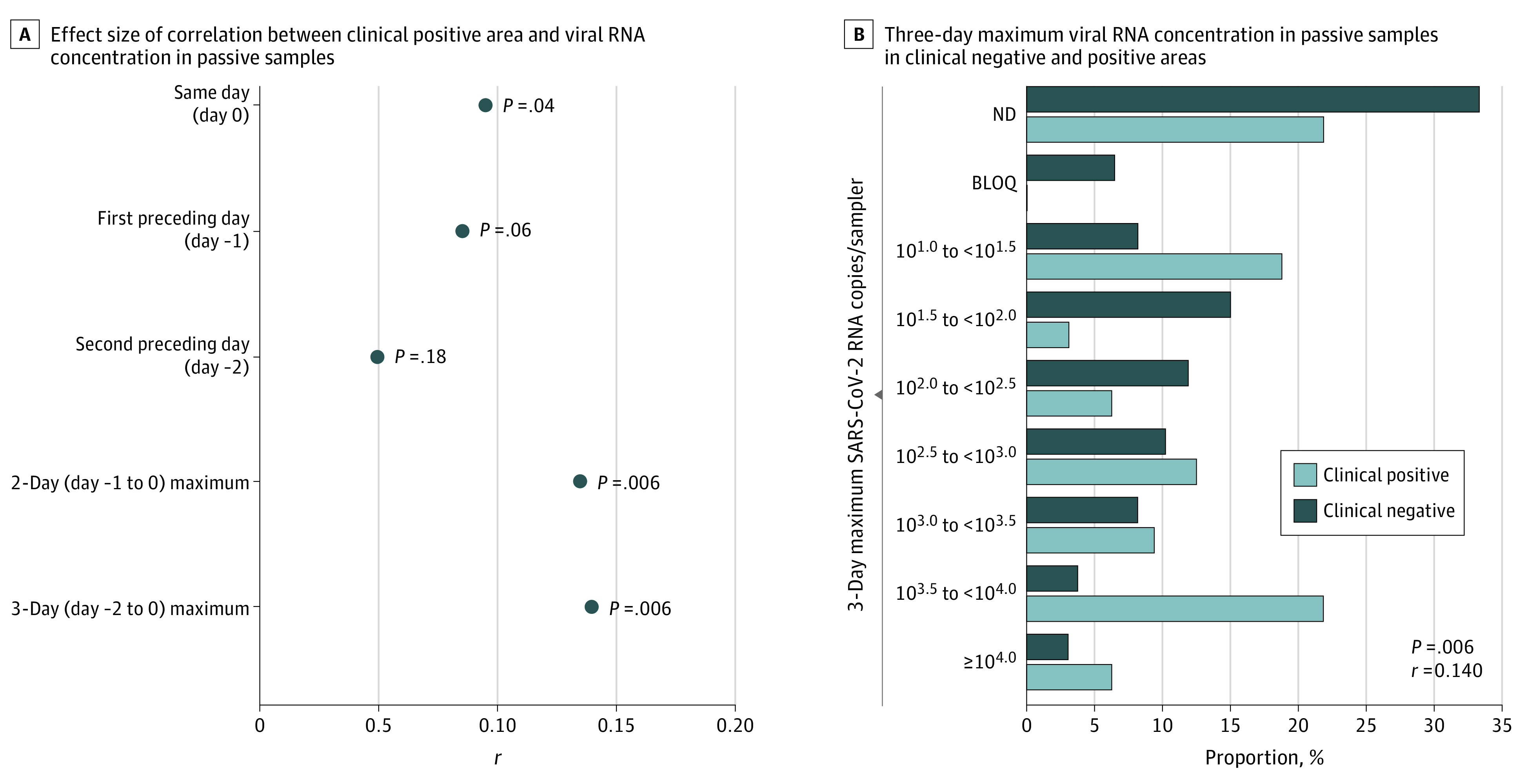
Correlation of SARS-CoV-2 RNA Load in Wastewater With Clinical Testing Results A, Correlation between the presence of areas where at least 1 confirmed COVID-19 case was reported (clinical positive area) and viral RNA concentrations observed in passive samples within the previous 3 days including the day when the clinical test results were obtained (day 0). B, Comparison of the 3-day maximum viral RNA concentrations in passive samples observed in clinical negative areas (no confirmed cases reported) and clinical positive areas. The 3-day maximum viral RNA concentration represents the maximum values in 3 consecutive days in which the last day was the day when corresponding clinical test results were obtained. One-tailed Mann-Whitney *U* test was used to investigate whether there was a statistically significant positive correlation between viral RNA load in passive samples and presence of clinical positive area. BLOQ indicates positive with below limit of quantification (<11 copies/sampler); ND, not detected.

The observed concentrations of SARS-CoV-2 RNA in passive samples were up to 35 000 copies per sampler. The strongest correlation between SARS-CoV-2 RNA load in wastewater and the presence of clinical positive area was found with 3-day (days −2 to 0) maximum wastewater concentrations (*r* = 0.140; *P* = .006, 1-tailed Mann-Whitney *U* test) (Figure 2A). Viral RNA load was positively correlated with presence of confirmed cases (Figure 2B).

## Discussion

Wastewater-based epidemiology (WBE) is a useful tool for detecting SARS-CoV-2 carriers at an early stage of transmission and monitoring distribution of the virus while protecting anonymity.^5^ However, additional tests for close contacts are essential for identifying and isolating individuals with potential infection and preventing further transmission. During the Tokyo Olympic and Paralympic Games, individuals with COVID-19 were quarantined outside the village.^3^ The correlation of SARS-CoV-2 RNA load in wastewater with the presence of clinical positive areas suggests that viral RNA was shed into sewers 2 days before the case was identified through clinical testing. Limitations of the study include the potential for trace amounts of viral RNA shed from an infected individual going undetected in wastewater and the numbers of participants in each area being unavailable.

The WBE data and other monitoring results (eg, number of positive cases, status of community transmission outside the village) reported to the organizing committee were collectively used as indicators of anticipated COVID-19 incidence,^1^ and enhanced infection prevention measures (eg, increased testing frequency for staff in physical contact with athletes) were implemented during the Paralympics.^6^ These findings suggest that WBE and clinical tests are complementary and that the testing strategy played a role in preventing COVID-19 clusters in the village. This study of one of the world’s largest mass gatherings provides novel evidence on the implementation and use of WBE in communities where all members undergo daily testing.

## Notes

Supplement.
eMethods.
