# Supplementary material for: Association of SARS-CoV-2 Load in Wastewater With Reported COVID-19 Cases in the Tokyo 2020 Olympic and Paralympic Village From July to September 2021
Source: JAMA Netw Open. 2022 Aug 22;5(8):e2226822. doi: 10.1001/jamanetworkopen.2022.26822 (PMC9396362; doi:10.1001/jamanetworkopen.2022.26822)
Supplement: Supplement. — eMethods. [file jamanetwopen-e2226822-s001.pdf]

Supplemental Online Content

Kitajima M, Murakami M, Kadoya SS, et al. Association of SARS-CoV-2 load in wastewater with reported COVID-19 cases in the Tokyo 2020 Olympic and Paralympic Village from July to September 2021. *JAMA Netw Open.* 2022;5(8):e2226822. doi:10.1001/jamanetworkopen.2022.26822

eMethods

This supplemental material has been provided by the authors to give readers additional information about their work.

© 2022 Kitajima M et al. *JAMA Network Open.*

eMethods

Wastewater sampling

Between July 14 and August 11, 2021 (Olympics period) and August 17 and September 8, 2021 (Paralympics period), 360 wastewater samples were collected from seven distinct areas (A to G) comprising of entire residential buildings in the Tokyo 2020 Olympic and Paralympic Village, via passive sampling from manholes. No wastewater sample was collected before July 14 (before Olympics) and August 12-16 (between Olympics and Paralympics) when the village was not open for athletes and staff. The residential buildings in the village were newly built and there was no resident (i.e., no domestic wastewater flow in the village) prior to the opening of the village, and therefore, no baseline test could be taken prior to the start of the Olympic period.

The passive samplers were made of cotton and rayon with a dimension of 66 mm × 50 mm. Each passive sampler was exposed to wastewater from one of the areas for 24 h, which was transported to the laboratory on ice and processed immediately upon arrival.

Viral RNA quantification

RNA extraction

Total RNA was extracted from the sampler using the RNeasy PowerWater kit (Qiagen, Hilden, Germany) according to the manufacturer's instructions with slight modifications. Briefly, the passive sampler was dewatered with a syringe, which was then subjected to RNA extraction with the kit to obtain a final RNA extract volume of 50 μL.

One-step RT-Preamplification

The RNA extract was subjected to one-step reverse transcription followed by pre-amplification (RT-Preamp) using the iScript™ Explore One-Step RT and PreAmp Kit (Bio-Rad Laboratories, Hercules, CA, USA) according to the manufacturer's instructions with slight modifications. Briefly, 13.5 μL of the RNA extract was mixed with 16.5 μL of the RT-Preamp reaction mixture containing 15.0 μL of SsoAdvanced Preamp Supermix, 0.6 μL of iScript Explore Reaction Booster, 0.6 μL of iScript Advanced Reverse Transcriptase, and 0.3 μL of primer mix containing 3 pmol each of CDC N1 forward (2019-nCoV_N1-F; 5'-GACCCCAAAATCAGCGAAAT-3') and reverse (2019-nCoV_N1-R; 5'- TCTGGTTACTGCCAGTTGAATCTG-3') primers[1] and 3 pmol of PMMoV reverse (PMMV_RP1; 5'-GAGTGGTTTGACCTTAACGTTTGA-3') primer[2]. The use of PMMoV reverse primer alone in this reaction terminates with reverse transcription for PMMoV RNA and prevents it from deviating from the quantitative range of qPCR for PMMoV, which is abundantly present in wastewater. The thermal cycling condition of RT-Preamp was 25°C for 5 mins, 45°C for 60 mins, and 95°C for 3 min followed by 10 cycles of 95°C for 15 s and 55°C for 4 min. Tenfold serial dilution of plasmid containing the amplification region sequence of the qPCR assay (CDC N1, Integrated DNA Technologies, Coralville, IA, USA) was included to generate a standard curve. Nuclease-free water was always used as a negative control in the RT-Preamp reaction.

qPCR

qPCR for SARS-CoV-2 was performed in a total reaction volume of 25 μL, which consisted of 2.5 μL of the RT-Preamp product and 22.5 μL of the qPCR reaction mixture. The latter contained 12.5 μL of the QuantiTect® Probe PCR Master Mix, 10 pmol each of CDC N1 forward (2019-nCoV_N1-F) and reverse (2019-nCoV_N1-R) primers, and 7.5 pmol of probe ((2019-nCoV_N1-P; 5'-FAM-ACCCCGCATTACGTTTGGTGGACC-BHQ1-3'). qPCR for PMMoV was performed in a total reaction volume of 25 μL, which consisted of 2.5 μL of the RT-Preamp product and 22.5 μL of the qPCR reaction mixture. The latter contained 12.5 μL of the QuantiTect® Probe PCR Master Mix, 10 pmol each of forward (PMMV_FP1-rev; 5'-GAGTGGTTTGACCTTAACGTTTGA-3')[3] and reverse (PMMV_RP1-rev) primers, and 7.5 pmol of probe (PMMV-Probe1; 5'-FAM-CCTACCGAAGCAAATG-NFQ-MGB-3') (Table 2). PCR amplification was performed with an StepOnePlus™ Real-Time PCR System (Thermo Fisher Scientific, Waltham, MA, USA) under the following conditions: 50°C for 2 min and initial denaturation at 95°C for 10 min to activate the DNA polymerase followed by 45 cycles of denaturation at 95°C for 3 s and annealing and extension at 55°C for 30 s. The thermal cycling conditions for SARS-CoV-2 and PMMoV assays

were identical. Nuclease-free water was used as a negative control in the qPCR reactions. Amplification data were collected and analyzed with Sequence Detector software version 2.0 (Thermo Fisher Scientific). The threshold value of relative fluorescent intensity (ΔRn) was adjusted to be 0.2 according to the manufacturer's (Qiagen) instructions for the qPCR master mix. Ten-fold serial dilutions of standard plasmid DNA for SARS-CoV-2 (10^1 to 10^5 copies per reaction) and gBlocks for PMMoV (10^1 to 10^7 copies per reaction) suspended in molecular-grade water was used for the quantification of viral copy numbers in the PCR tubes. The slope (S) of the linear regression curve correlates with efficiency (E) of the PCR according to the formula $E = 10^{-1/S} - 1$. Cut-off threshold cycle (Ct) was set at 40 and qPCR results were carefully interpreted according to the Minimum Information for Publication of Quantitative Real-Time PCR Experiments (MIQE) guidelines[4,5]. PMMoV RNA was measured as a fecal strength indicator and an internal control to validate the results of SARS-CoV-2 RNA detection.

Ethics statement

The wastewater data obtained in the present study did not contain any personal information (i.e., anonymous data) and the data on the numbers of confirmed cases and close contacts tests were non-personally identifiable and publicly available from the Organising Committee and Akashi et al. (2022)[6], respectively. Therefore, informed consent and ethics committee approval were not required because this study was outside the scope of the guidelines set by the Ministry of Education, Culture, Sports, Science and Technology, Japan ("Ethical Guidelines for Medical and Biological Research Involving Human Subjects")[7]. This study followed the reporting requirements of the Strengthening the Reporting of Observational Studies in Epidemiology (STROBE) Statement.[8]

Supplemental References

1. Centers for Disease Control and Prevention. 2019-novel coronavirus (2019-nCoV) real-time rRT-PCR panel primers and probes. Updated June 6, 2020. Accessed February 6, 2022. https://www.cdc.gov/coronavirus/2019-ncov/lab/rt-pcr-panel-primer-probes.html
2. Zhang T, Breitbart M, Lee WH, Run JQ, Wei CL, Soh SWL, Hibberd ML, Liu ET, Rohwer F, Ruan Y. RNA viral community in human feces: Prevalence of plant pathogenic viruses. PLoS Biology. 2006;4:0108–0118. doi:10.1371/journal.pbio.0040003
3. Haramoto E, Kitajima M, Kishida N, Konno Y, Katayama H, Asami M, Akiba M. Occurrence of pepper mild mottle virus in drinking water sources in Japan. Applied and Environmental Microbiology. 2013;79:7413–7418. doi:10.1128/AEM.02354-13
4. Bustin SA, Benes V, Garson JA, Hellemans J, Huggett JF, Kubista M, Mueller RD, Nolan T, Pfaffl MW, Shipley GL, Vandesompele J, Wittwer CT. The MIQE guidelines: Minimum information for publication of quantitative real-time PCR experiments. Clin. Chem. 2009;55:611–622. doi:10.1373/clinchem.2008.112797
5. Bivins A, Kaya D, Bibby K, Simpson SL, Bustin SA, Shanks OC, Ahmed W. Variability in RT-qPCR assay parameters indicates unreliable SARS-CoV-2 RNA quantification for wastewater surveillance. Water Res. 2021;203:117516. doi:10.1016/j.watres.2021.117516
6. Akashi H, Shimada S, Tamura T, Chinda E, Kokudo N. SARS-CoV-2 infections in close contacts of positive cases in the Olympic and Paralympic Village at the 2021 Tokyo Olympic and Paralympic Games [published online February 3, 2022]. JAMA. doi:10.1001/jama.2022.0818
7. Ministry of Education, Culture, Sports, Science and Technology. Ethical Guidelines for Medical and Biological Research Involving Human Subjects [in Japanese; published March 23, 2021, revised March 10, 2022]. https://www.mhlw.go.jp/content/000909926.pdf
8. von Elm E, Altman DG, Egger M, Pocock SJ, Gøtzsche PC, Vandenbroucke JP; STROBE Initiative. The Strengthening the Reporting of Observational Studies in Epidemiology (STROBE) statement: guidelines for reporting observational studies. Prev Med. 2007;45(4):247-251. doi:10.1016/j.ypmed.2007.08.012

© 2022 Kitajima M et al. *JAMA Network Open.*
